# Revisiting the role of light signaling in plant responses to salt stress

**DOI:** 10.1093/hr/uhae262

**Published:** 2024-09-16

**Authors:** Yinxia Peng, Haiyan Zhu, Yiting Wang, Jin Kang, Lixia Hu, Ling Li, Kangyou Zhu, Jiarong Yan, Xin Bu, Xiujie Wang, Ying Zhang, Xin Sun, Golam Jalal Ahammed, Chao Jiang, Sida Meng, Yufeng Liu, Zhouping Sun, Mingfang Qi, Tianlai Li, Feng Wang

**Affiliations:** College of Horticulture, Shenyang Agricultural University, Shenyang 110866, China; Key Laboratory of Protected Horticulture, Ministry of Education, Shenyang 110866, China; National & Local Joint Engineering Research Center of Northern Horticultural Facilities Design & Application Technology (Liaoning), Shenyang 110866, China; College of Horticulture, Shenyang Agricultural University, Shenyang 110866, China; College of Horticulture, Shenyang Agricultural University, Shenyang 110866, China; College of Horticulture, Shenyang Agricultural University, Shenyang 110866, China; College of Horticulture, Shenyang Agricultural University, Shenyang 110866, China; College of Horticulture, Shenyang Agricultural University, Shenyang 110866, China; College of Horticulture, Shenyang Agricultural University, Shenyang 110866, China; College of Horticulture, Shenyang Agricultural University, Shenyang 110866, China; College of Horticulture, Shenyang Agricultural University, Shenyang 110866, China; College of Horticulture, Shenyang Agricultural University, Shenyang 110866, China; College of Horticulture, Shenyang Agricultural University, Shenyang 110866, China; College of Land and Environment, Shenyang Agricultural University, Shenyang 110866, China; College of Horticulture and Plant Protection, Henan University of Science and Technology, Luoyang 471023, China; School of Agriculture, Hulunbuir University, Hulunbuir 021008, China; College of Horticulture, Shenyang Agricultural University, Shenyang 110866, China; College of Horticulture, Shenyang Agricultural University, Shenyang 110866, China; College of Horticulture, Shenyang Agricultural University, Shenyang 110866, China; College of Horticulture, Shenyang Agricultural University, Shenyang 110866, China; College of Horticulture, Shenyang Agricultural University, Shenyang 110866, China; Key Laboratory of Protected Horticulture, Ministry of Education, Shenyang 110866, China; National & Local Joint Engineering Research Center of Northern Horticultural Facilities Design & Application Technology (Liaoning), Shenyang 110866, China; College of Horticulture, Shenyang Agricultural University, Shenyang 110866, China; Key Laboratory of Protected Horticulture, Ministry of Education, Shenyang 110866, China; National & Local Joint Engineering Research Center of Northern Horticultural Facilities Design & Application Technology (Liaoning), Shenyang 110866, China

## Abstract

As one of the grave environmental hazards, soil salinization seriously limits crop productivity, growth, and development. When plants are exposed to salt stress, they suffer a sequence of damage mainly caused by osmotic stress, ion toxicity, and subsequently oxidative stress. As sessile organisms, plants have developed many physiological and biochemical strategies to mitigate the impact of salt stress. These strategies include altering root development direction, shortening the life cycle, accelerating dormancy, closing stomata to reduce transpiration, and decreasing biomass. Apart from being a prime energy source, light is an environmental signal that profoundly influences plant growth and development and also participates in plants' response to salt stress. This review summarizes the regulatory network of salt tolerance by light signals in plants, which is vital to further understanding plants' adaptation to high salinity. In addition, the review highlights potential future uses of genetic engineering and light supplement technology by light-emitting diode (LED) to improve crop growth in saline–alkali environments in order to make full use of the vast saline land.

## Introduction

Soil salinization is a global issue that threatens vegetable growth and harvest, particularly in protected cultivation systems, and hinders the sustainable progress of contemporary agriculture [1]. More than one-third of irrigated land is deteriorated by salinization. With the unprecedented extreme climate, improper drainage and irrigation systems, and some other factors, the problem of cultivated land salinization has further increased, seriously threatening food security [1]. Salt stress inhibits vegetable growth and even causes death, quality deterioration, and yield decline [2]. Therefore, it is critical to explore how plants respond to high salinity.

**Figure 1:**
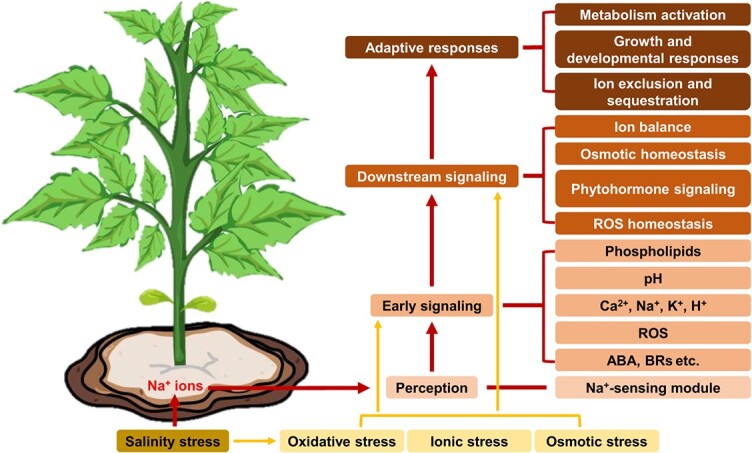
Salt stress accompanied with oxidative stress, ionic stress and osmotic stress cause various physiological and molecular changes in plants. After receiving sodium ion input, plants generate some early signals, which are transferred to the downstream for some physiological and biochemical changes to produce some adaptive responses like metabolism activation, growth and developmental responses, ion exclusion, and sequestration. ROS, reactive oxygen species; ABA, abscisic acid; BRs, brassinosteroids.

Salt stress rapidly triggers several secondary damages causing ionic stress, oxidative stress, and osmotic stress [3–5]. After receiving an abundance of sodium ion input, plants generate some early signals, which are transferred downstream for some physiological and biochemical changes to produce some adaptive responses, including metabolic adjustment, ion sequestration, and exclusion [6] (Fig. 1). For example, plants maintain the proportion of ions to water in balance by keeping high water content, avoiding Na^+^ accumulation in the shoots by ion exclusion, and subsequent sequestration in vacuoles [7], altering the direction of root development [3, 7], shortening the life cycle, and accelerating the entry into dormancy [4]. They also mitigate the damage of salt stress by closing stomata to reduce transpiration and reducing biomass [5].

Recently, some studies have suggested that light is also essential for plant responses to salt stress [1]. In addition to being a vital source of energy, light also serves as a critical regulator of plant replies to various stresses as an environmental signal [1]. Variations in light quality and intensity, as well as light duration, are the primary factors affecting plant tolerance to salt stress [1]. Recently, it has been reported that salt stress-induced SALT OVERLY SENSITIVE 2 (SOS2) kinase activity is significantly boosted by light-activated photoreceptors, PHYTOCHROME A (phyA) and phyB [8]. Understanding the mechanism of light-mediated plant responses to salt stress is of great significance to ensure crop quality and improve yield. Here, we present current advances in clarifying the mechanism of light signaling-mediated plant responses to salt stress.

## Sensing and response to salt stress

Salt stress has a deleterious impact on plant growth stages and it impedes plant development [9]. High salt concentrations in plant cells can induce the first constraint: osmotic stress, which reduces the ability of plants to uptake water [10]. Meanwhile, the levels of toxic ions sodium (Na^+^) and chloride (Cl^−^) steeply increase and this process triggers disruption of ion homeostasis [11]. The accumulation of water-soluble salts such as Na^+^, Cl^−^, potassium (K^+^), and sulfate (SO_4_^2−^) in the soil at the root of the plant leads to ionic toxicity [3, 12, 13]. High pH in alkaline saline soil dominated by sodium carbonate (Na_2_CO_3_) and sodium bicarbonate (NaHCO_3_) reduces the Na^+^ exclusion [14], resulting in high cellular oxidative stress compared with salinity alone [8, 13] (Fig. 1). Salt stress hinders plant growth accompanied by various physiological and molecular changes [3–5]. In particular, salt stress inhibits photosynthesis, thereby reducing the resources accessible and obstructing cell division as well as expansion [15]. Salt stress also affects the formation of light-trapping complexes that regulate shifts in the photosynthetic state [16]. Regulation of glycosylation under saline stress conditions can change the activity or protein stability of essential photosynthetic enzymes such as RIBULOSE-1,5-BISPHOSPHATE CARBOXYLASE/OXYGENASE (RuBisCO) [17]. In addition, salt stress regulates sugar signaling by changing sucrose and fructose levels, and glycolytic processes [17, 18].

**Figure 2:**
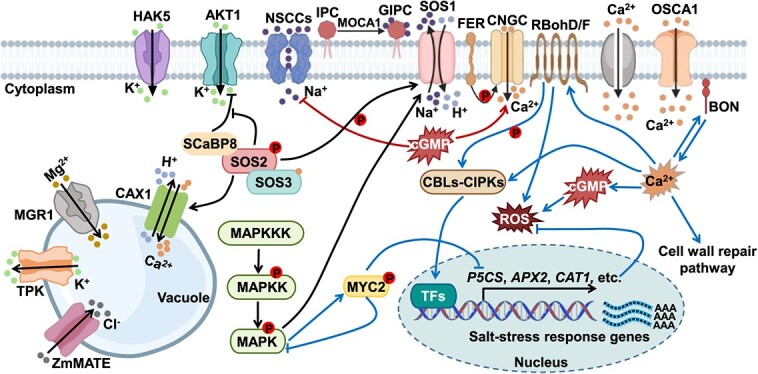
Plant salt response signaling. Sodium ions (Na^+^) enter the cell through NSCC mechanisms. After attaching to sodium ions, GIPC increases Ca^2+^ intake, whereas MOCA1 inhibits its activity. At the same time, several ion channels are activated due to membrane depolarization. Salt stress activates Ca^2+^ channels OSCA1 to generate specific calcium waves thereby activating the CBLs–CIPKs pathway and ROS signaling, transcribing downstream salt-stress responsive genes, ROS scavenging system, and proline in response to salt stress. The cGMP bursts increase salt tolerance by inhibiting Na^+^ input and promoting Ca^2+^ input. SOS1/SCaBP8 is activated by Ca^2+^, which activates the SOS pathway. Simultaneously, SOS2 increases the activity of the vesicular membrane channel protein CAX1 and blocks the inhibitory impact of ScaBP8 on the K^+^ channel AKT1. The K^+^ channel opens, and the influx of K^+^ promotes ionic equilibrium. MGR1 mediates Mg^2+^ entry into the vacuole, while ZmMATE29 promotes shoot Cl^−^ exclusion likely by compartmentalizing Cl^−^ into the vacuoles of root cortex cells. Additionally, the MAPK cascade is activated by stepwise phosphorylation, and active MAPK6 stimulates SOS1 to regulate ion homeostasis under salt stress. MAPK6 also contributes in the regulation of salt stress through the participation of MYC2 in the synthesis of proline. Plants mainly improve salt tolerance from ion balance and oxidative regulation.

Early cues that stimulate the salt stress response include elevated intracellular Ca^2+^ levels, excessive accumulation of Na^+^ in the extracellular plastid, 3,5-CYCLIC GUANOSINE MONOPHOSPHATE (cGMP) production, REACTIVE OXYGEN SPECIES (ROS) accumulation, and the induction of these early signals initiates a series of complex signal network of plants [19, 20] (Fig. 1). The onset of salt stress leads to a transient and rapid increase in intracellular Ca^2+^, which triggers a calcium signaling cascade by binding to and activating calcium ion receptors in a few seconds [21] (Fig. 2). Changes in salt stress and osmotic stress are relevant to the activation of calcium channels, and the plasma membrane calcium channel REDUCED HYPEROSMOLALITY-INDUCED [Ca^2+^]cyt INCREASE1 (OSCA1) is thought to be required for osmotic stress to induce this signal transduction [22, 23]. OSCA1 generates specific calcium waves to activate the CALCINEURIN B-LIKE AND CBL-INTERACTING PROTEIN KINASE (CBL-CIPK) pathway, transcribing downstream salt-stress responsive genes [22, 23]. At the same time, the SOS pathway is also activated by Ca^2+^ [22, 23]. The three components of the evolutionarily conserved SOS signaling pathway are the calcium-binding protein (SOS3), protein kinase (SOS2), and plasma membrane Na^+^/K^+^ antiporter (SOS1), which are critical for salt tolerance [24]. SOS3/SOS3-LIKE CALCIUM-BINDING PROTEIN 8 (SCaBP8) catches the second messenger Ca^2+^ and activates it to phosphorylate members of the SOS pathway in a stepwise manner, ultimately activating SOS1 [15], which removes Na^+^ from the cell and increases salt tolerance (Fig. 2). Furthermore, SOS2 activates the vesicular counter-transporter protein CATION EXCHANGER 1 (CAX1), which regulates ion homeostasis [25] (Fig. 2).

MONOCATION-INDUCED [Ca^2+^]cyt INCREASES 1 (MOCA1) could improve salt resistance in *Arabidopsis* as an Na^+^-gated calcium channel [26]. MOCA1 is involved in GLYCOSYL INOSITOL PHOSPHORYLCERAMIDE (GIPC) biosynthesis by encoding glucuronosyltransferase (Fig. 2). GIPC inversely activates MOCA1 to increase stress-induced Ca^2+^ influx, whereas extracellular Na^+^ can bind GIPC to regulate salt stress responses [26]. High Na^+^ level also competes with K^+^ absorption, which is involved in a number of physiological processes in plants [27]. Since the soil has negligible K^+^ content and K^+^ is beneficial in promoting plant growth under salt stress, the cytoplasmic Na^+^/K^+^ ratio is another crucial marker of crop salt tolerance [28]. TONOPLAST K^+^-PERMEABLE CHANNEL (TPK) regulates Na^+^/K^+^ homeostasis by pumping K^+^ from the vacuolar into the cytoplasm to improve salt tolerance in plants [29]. Numerous mineral elements (e.g. magnesium, nitrogen, phosphorus, and potassium), which are closely linked to photosynthesis, protein synthesis, and energy storage and transfer, are also in competition with the major salt stress ions, Na^+^ and Cl^−^ [30–32]. In addition, Mg^2+^ transported by MAGNESIUM TRANSPORTER (OsMGT1) enhances salt tolerance via HIGH-AFFINITY K^+^ TRANSPORTERS (OsHKT1;5)-mediated xylem Na^+^ unloading in roots [33]. Plants also have ACDP-TYPE TRANSPORTER 1 (MGR1), which mediates Mg^2+^ sequestration into the vacuole to detoxify excess Mg^2+^ in the cytoplasm [34] (Fig. 2). The MULTIDRUG AND TOXIC COMPOUND EXTRUSION (ZmMATE29) promotes shoot Cl^−^ exclusion likely by compartmentalizing Cl^−^ into the vacuoles of root cortex cells [35].

Recent studies have found that ABSCISIC ACID (ABA) accumulation, calcium-responsive phospholipid-binding BONZAI (BON) proteins, and related-pathway gene expression are all positively regulated by osmotic stress, which suggests that BON proteins may be a critical regulator of osmotic stress perception and signal transduction [36] (Fig. 2). Interestingly, the plasma-membrane-localized receptor kinase FERONIA (FER) has been revealed to be necessary for the preservation of cell wall integrity and the restoration of root growth [37]. FER interacts with cell wall component pectin to sense the cell wall malacia by salinity [37] (Fig. 2). In addition, CYCLIC NUCLEOTIDE-GATED CHANNELs (CNGCs) are subject to intracellular calcium concentration inhibition and regulation by the calcium channel proteins, such as CALMODULIN (CaM) [36]. Meanwhile, FER also phosphorylates CNGCs to regulate calcium [38, 39] (Fig. 2). However, how plant cells perceive the sodium ion signal in the early stage and response to salt stress remains unclear. Excess Na^+^ can enter plant root cells through NON-SELECTIVE CATION CHANNELS (NSCCs) (Fig. 2), which transport sodium ions through the plasma membrane [40]. Thus, early salt-induced signals may be involved in regulating NSCCs during plant response. CNGCs are the main form of NSCCs involved in ion homeostasis during intracellular salt response [41]. Other transporters and channels may also work in the perception of salinity, but their specific roles are unknown [41].

Intracellular cGMP levels are elevated in a matter of seconds following the onset of salt stress [42]. It has been shown that cGMP inhibits Na^+^ uptake in *Arabidopsis* and pepper [43, 44], while promoting the absorption of K^+^ uptake [45, 46]. The cGMP negatively regulates Na^+^ uptake through regulating NSCCs [47]. Interestingly, cyclic cGMP and calcium mediate phytochrome phototransduction and target distinct phytochrome-responsive elements [48, 49], which indicates that phytochrome A may require cGMP to enhance anthocyanin synthesis and chlorophyll accumulation to respond to salt stress [48, 49]. In addition, a reduction in the cytosolic cGMP is essential for UV-A radiation-inhibited stomatal opening, which is mediated by a cGMP-ACTIVATED PHOSPHODIESTERASE (AtCN-PDE1) [50]. The transcriptional expression of *CmHKT1;1* and *CORNICHON HOMOLOG* (*CmCNIH1*) in the root of cucumber was obviously increased when salt stress occurred [51]. Consequently, CmCNIH1 specifically promotes the deposition of CmHKT1;1, which is located on the membrane, by facilitating the endoplasmic reticulum-to-Golgi apparatus secretory pathway or from Golgi apparatus-to-plasma membrane [51]. The high Na^+^ content in the xylem parenchyma cells inhibits Na^+^ transport from the roots to the aboveground, thereby increasing salt tolerance in cucumber [51]. In addition, the MITOGEN-ACTIVATED PROTEIN KINASE (MAPK) cascade is essential to respond to osmotic stress [52, 53]. Salt stress activates MKK4, then promotes MPK3 accumulation, which induces the gene expression of *NINECIS-EPOXYCAROTENOID DIOXYGENASE 3* (*NCED3*) and *RESPONSIVE TO DESSICATION 29A* (*RD29A*) [53]. The ROS level and salt sensitivity are increased after *Atmkk4* mutation [53]. Moreover, the salt tolerance is significantly increased after overexpressing the *AtMAPKKK20* gene [52] (Fig. 2). Interestingly, salt stress induces phosphatidic acid (PA) accumulation [54]. PA increases salt tolerance via binding to MPK6 and stimulating SOS1 phosphorylation (Fig. 2), then enhancing the Na^+^ exclusion ability of SOS1 [54].

ROS are essential for calcium signaling activation. It has been shown that ROS generated in the RESPIRATORY BURST OXIDASE HOMOLOG D (RBOHD) diffuse through the apoplast [55] (Fig. 2). The leucine-rich repeat receptor kinase HYDROGEN-PEROXIDE-INDUCED CA^2+^ INCREASES1 (HPCA1) increases the concentration of Ca^2+^ in the cytoplasm upon sensing H_2_O_2_ [56]. The increased Ca^2+^ wave activates more RBOHD proteins [55]. Furthermore, vascular Na^+^ concentration in the root vascular system is regulated by *RBOHF*-dependent ROS buildup, which in turn influences vascular Na^+^ concentration in shoots [57]. *AtRBOHF* loss of function under salt stress conditions increases root vascular system and xylem Na^+^ levels, thereby increasing Na^+^ accumulation in shoots through transpiration, which leads to higher salt sensitivity [58]. Moreover, Ca^2+^ concentration activated the CALCINEURIN B-LIKE PROTEIN-CBL1/9-INTERACTING PROTEIN KINASE (CBL1/9-CIPK26) signaling pathway activity to interact with and phosphorylate AtRBOHF [59] (Fig. 2). Interestingly, the nuclear localization protein CONSTANS HOMOLOG 1a (GmCOL1a) protects soybean plants from salt stress injury by directly activating *Δ^1^-PYRROLINE-5 CARBOXYLATE SYNTHETASE* (*GmP5CS*) gene expression to promote proline accumulation [60]. In addition, GmCOL1a may also directly inhibit ROS production through the *GmP5CS*-independent pathway [60]. Thus, ROS and Ca^2+^ signaling act synergistically to influence cellular pH and ion homeostasis during the early stages of salt stress.

## The role of light signaling in plant response to salt stress

One of the key environmental signals for regulating the growth and development of plants is light. Photoreceptors receive light signals and interact with downstream signaling factors such as E3 ubiquitin ligase complex CONSTITUTIVELY PHOTOMORPHOGENIC 1 (COP1), PHOTOCHROME INTERACTING FACTORS (PIFs), and SUPPRESSOR OF phyA-105 (SPA), which in turn regulate the expression of downstream genes in response to shifts in environmental signals [61, 62]. COP1 destabilizes the stability of multiple positive TRANSCRIPTION FACTORS (TFs) in the dark and plays an inhibitory role in the optical signal network [63]. Under light conditions, activated PHYs and CRYPTOCHROMES (CRYs) repress COP1/SPA expression [64, 65]. The Pfr activated at the initial stage of light exposure binds to the COP1/SPA complex after entering the nucleus, forcing the complex to recombine, leading to inactivation of the enzymatic activity of the E3 ubiquitin ligase of COP1 [62]. Additionally, reports have indicated that the decline of COP1 activity is due to the induction of COP1 efflux from the nucleus by phytochrome and that COP1 no longer binds to SPA, thus stabilizing a large number of TFs that positively regulate photomorphogenesis [66]. The COP1/SPA complex degrades many light signaling factors via the 26S proteasome pathway, including LONG HYPOCOTYL IN FAR-RED 1 (HFR1), ELONGATED HYPOCOTYL 5 (HY5), B-BOX CONTAINING PROTEINS (BBXs), HY5-HOMOLOG (HYH), LONG AFTER FAR-RED LIGHT1 (LAF1), and HECATEs (HECs) [63, 67–73]. The exposure to salt stress induces the translocation of COP1 from the nucleus to the cytoplasm, consequently attenuating downstream protein degradation and thereby participating in the regulation of salt tolerance [74]. The accumulation of HY5 protein directly binds to promoters of *P5CS1* and *ABSCISIC ACID-INSENSITIVE 5 (ABI5)* genes to activate their transcription, which induces proline and ABA accumulation, thereby enhancing plant salt tolerance [75]. However, ethylene (ET) facilitates the nuclear import of COP1, counteracting its nucleocytoplasmic shuttling induced by salt stress and establishing a complex regulatory network [74]. A BBX protein, SALT TOLERANCE PROTEIN (STO), which is inhibited by COP1, negatively regulates hypocotyl growth under blue light signaling [76]. HIGH-AFFINITY K^+^ TRANSPORTER (HAK) family transporters play a crucial role in plant tolerance to salt stress [77]. Interestingly, OsBBX17 can bind to the *OsHAK2/7* promoter and repress its transcriptional level in rice [75]. Salt stress inhibits *OsBBX17* expression, but activates MAPK1 [75]. The phosphorylation of OsBBX17 by OsMAPK1 further inhibits its repression of *OsHAK2/7*, promoting ion homeostasis and salt tolerance [75]. In addition, red light induces phosphorylation of MKK2-MPK2 in guard cells, which is dependent on phyB [78]. Activated MPK2 affects the expression of ABA-signaling genes and fine-tunes stomatal movement [78], thereby regulating plant salt tolerance.

PIFs belong to the basic HELIX–LOOP–HELIX (bHLH) family [79–81]. As the fundamental inhibitory factor in the process of photomorphogenesis, PIFs are accumulated in the dark environment [82–85]. The activated phytochrome enters the nucleus to phosphorylate and degrade PIFs, relieving their inhibitory effects [82–85]. In addition, activated Pfr interacts with PIF in the nucleus and inhibits its activity [85–87]. It has been shown that AtPIF1/3 exerts a negative regulatory effect on salt tolerance by inhibiting the transcription of downstream genes [8]. In addition, PIF reduces salt tolerance by suppressing ethylene synthesis [88], which is a positive regulator of plant salt tolerance [89]. However, silencing the *CaPIF8* gene in pepper plants also exhibits a salt-sensitive phenotype [90], which indicates that PIFs may have different functions in a variety of plants. Studies have shown that light signals, such as light intensity, light quality, and photoperiod, play an indispensable role in plant response to salt stress through regulating photoreceptors and a lot of light signaling factors [28].

## Light intensity regulation of salt tolerance

In nature, light intensity changes continuously with day–night and seasonal changes. The transcriptional activation of PIFs promotes the elongation of plant hypocotyls under low-light conditions [79–81]. It has been shown that under salt stress conditions, the ABA signaling pathway inhibits the PIF-BES1 (BRI1-EMS-SUPPRESSOR1) module by suppressing the expression of *BRASSINOSTEROID-SIGNALING KINASE 5* (*BSK5*) [91]. Salt stress antagonizes the shade-induced elongation of hypocotyls [91]. Furthermore, a recent study has demonstrated that the germination rate of seeds is proportional to the rise with increasing light intensity when exposed to salt stress [92]. The main reason is that light promotes the accumulation of DELLA protein during seed germination under salt stress [92]. Hence, the combined activity of light intensity and DELLA proteins under salinity stress could be exploited to enhance crop productivity.

## Light quality regulation of salt tolerance

Plants are able to adapt to changing light quality through morphological, physiological, and biochemical changes [93]. Salt stress slows the electron transfer from REACTION CENTERS (RCs) to plastid quinones [94], interferes with the electron transfer by affecting the electron transfer chain on both the donor and acceptor sides, and reduces the photosynthetic efficiency of plants [95]. The buildup of NaCl in mesophyll cells causes a decrease in carbon uptake and a rise in internal CO_2_ concentration, leading to a significant decrease in the plant's stomatal conductance [96]. Stomatal closure is a critical factor in the reduction of photosynthesis in plants under moderate or high salt stress [97, 98]. It was extensively researched and documented that blue light not only promotes photosynthesis of plants, but also indirectly affects the opening of stomata and increases transpiration [99, 100]. The response of chloroplast guard cells to red light and the reaction of stomatal guard cells to decreased intercellular CO_2_ concentration cause the stomatal opening under red light [100]. Salt stress inhibits photosynthesis through reducing stomatal opening and carbon uptake, and disruption of photochemical reactions [101]. A previous study has shown that adding some blue light in red light significantly affects the amount of CO_2_ uptake under salinity stress conditions [102]. In addition, a G-BOX BINDING FACTOR 1 (TaGBF1), which is induced by blue light, confers hypersensitivity to ABA and salt in wheat and *Arabidopsis* [103]. Interestingly, low R:FR light inhibits endogenous H_2_O_2_ by modulating the expression of *RBOH* genes, and simultaneously promotes the opening of stomata to enhance the photosynthetic rate of leaves, thus improving the salt tolerance in tomato [104]. Salt stress suppresses the PIF-BES1 signaling module to restrain the elongation of hypocotyl, but reducing the ratio of R:FR light could relieve the suppression of salt stress by enhancing the stability of PIF4, PIF5, and PIF7 protein [91]. These results suggest that the impacts of salinity stress can be reduced by manipulating the additional light spectrum.

## Photoperiod regulation of salt tolerance

Salt tolerance in plants is also correlated with photoperiod, and plants are more vulnerable to salt stress during the day than at night [19]. Salt-induced gene expression of *RD29A* and protein abundance of SOS1 showed a strong oscillation during a light–dark cycle [19], suggesting that plant salinity tolerance is photoperiodically regulated [105]. The core component of the circadian clock, GIGANTEA (GI), can coordinate flowering time through the typical GIGANTEA-CONSTANS-FLOWERING LOCUS T (GI-CO-FT) pathway [106, 107], as well as through the SOS pathway to adapt to salt stress [108]. In a normal state, GI interacts with SOS2 and prevents SOS1 phosphorylation and activation by SOS2 [108]. After salt stress, the E3 ubiquitin ligase COP1 transfers from the nucleus to the cytoplasm, and degrades the GI [109], which in turn releases SOS2. Plant salt tolerance is increased when SOS2 and SOS3 combine to create a complex that phosphorylates and activates SOS1 [108]. EARLY FLOWERING 3 (ELF3) is another essential component of the circadian clock, which acts as a connector between ELF4 and LUX ARRHYTHMIA (LUX) [110–112]. They form the EVENING COMPLEX (EC) to maintain a stable circadian rhythm and regulate salt tolerance [110–112]. ELF3 enhances salt tolerance by inhibiting *PIF4* transcription and inducing GI degradation [105]. In addition, *OsELF4a* is rapidly induced by salt stress and positively regulates salt tolerance in rice [113]. Interestingly, other core clock components, such as PSEUDO-RESPONSE REGULATOR 5, 7, and 9 (PRR5,7,9) also negatively regulate plant salt tolerance [114, 115]. These results indicate the genetic capacity of the circadian clock to maintain timekeeping activity over a broad range of salinity levels. These circadian core component gene mutants could be an ideal material for future molecular breeding.

## The effects of hormones on light-mediated salt stress pathways

The cross-talk pathway between light signaling and salt stress stimuli has been investigated recently. A previous study reported that *Nicotiana tobacum* resistance to salt stress is negatively regulated by phytochrome A and B through ABA-jasmonate acid (JA) synergistic cross-talk [116], but further research revealed that *Arabidopsis* exhibited salt-sensitive phenotypes after the mutation of *phyA*, *phyB*, or *phyAB* in light condition [8]. In addition, turf grass’s resistance to high saline soil is strengthened by the heterologous overexpression of a hyperactive mutant of oat *phyA* [8]. Interestingly, both photo-activated phyA and phyB positively control salt tolerance by cooperating with SOS2 protein and increasing SOS2 protein kinase activity without increasing its protein abundance [8]. On the other hand, phyA is activated while phyB is deactivated under deep shadow conditions [8]. Thus, it will be interesting to look into how varied shade situations affect the strategies of plants’ adaptations to salt stress.

As soil salinization increases and the total amount of cultivable land is predicted to decrease globally, fields are often intensively planted in order to obtain higher yields. Plant shade appears as a result of dense planting [8]. Shade reduces the ratio of red to far-red (R/FR) light in the light composition, and activates the gene expression to induce hormone synthesis [such as auxin, brassinosteroid (BR)] through regulating PIF proteins [117], which facilitates cell wall relaxation and plant growth [117]. In *Arabidopsis*, the low ratio of R/FR stimulates the expression of *PIF4*, *PIF5*, and *PIF7* [91]. The high transcript levels of these TFs also stimulate the transcriptional activation of downstream *BSK5*, which represses Glycogen synthase kinase 3-like kinase, leading to the activation of the BES1-PIF4/PIF5 module, thus promoting hypocotyl elongation [91]. Studies have demonstrated that low soil NaCl concentrations can lead to a reduction in plant biomass accumulation of stem and root, and reduce the plants' capacity to react to shade [118]. After salt stress, the ABA signaling pathway is activated [91]. The ABA-RESPONSIVE ELEMENT BINDING PROTEIN/FACTOR (AREB/ABF) transcription factor inhibits the *BSK5* transcription level, which in turn mediates the low R/FR-induced PIF-BES1 pathway and suppresses plant shade response [91].

**Figure 3:**
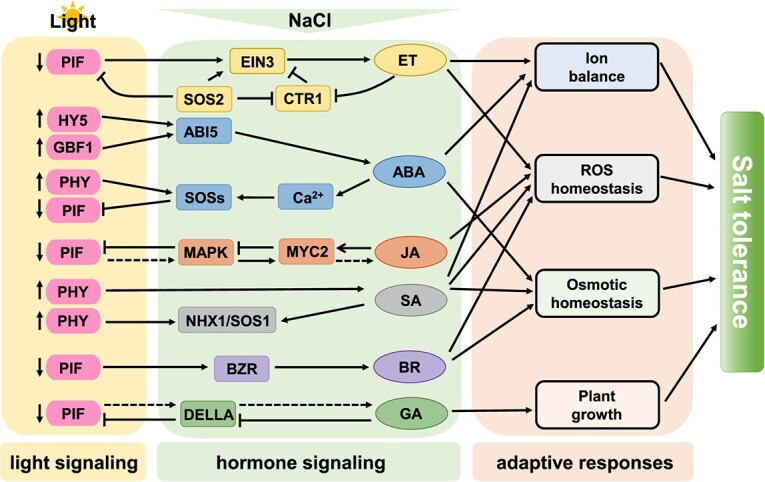
Hormone-mediated responses to light signaling and salt stress. Under normal conditions, the receptors of ethylene bind to and activate the negative regulator of ethylene signaling, CONSTITUTIVE TRIPLE RESPONSE1 (CTR1). Under salt stress, plants emanate ethylene gas, which activates EIN3 accumulation. PIFs encourage EIN3 accumulation and stimulate ethylene production to enhance salt tolerance by regulating ion homeostasis and scavenging ROS. Light-activated HY5 and GBF1 promote the large synthesis of ABA through ABI5, which promotes calcium wave generation and activates the SOS pathway to enhance salt tolerance. Salt stress induces the expression of JA biosynthesis-related genes in leaves and roots, leading to increased JA production. The crucial component activating JA signaling, MYC2, contributes to salt tolerance by regulating the proline biosynthesis gene. Light can also stimulate the synthesis of SA, which increases plant salt tolerance by activating the ion channels NHX1/SOS1 for ion balancing. BR can relieve salt toxicity by regulating the activity of Na^+^/H^+^ antiporters and NHX, and inhibiting ROS generation. BZR1, a BZR/BES TF, can interact with PIFs to positively regulate BR signaling and salt stress tolerance. In order to fine-tune plant development under salt stress, OsPIL14 and OsSLR1 (a DELLA protein) build a transcriptional module that integrates light and GA signals. DELLA starts a feedback loop that controls GA production in response to salt stress.

**Figure 4:**
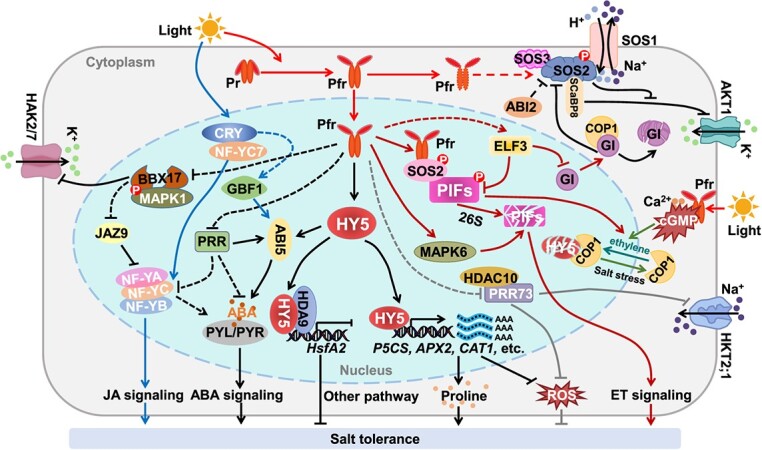
Light signaling regulates plants’ salt tolerance. The light signal promotes conformational changes of the phytochrome molecule from its Pr to active Pfr form, which enter the nucleus to promote the kinase activity of SOS2. SOS2 interacts with and phosphorylates SOS1 to activate it extruding Na^+^ from the cytosol to the apoplast. In addition, SOS2 activity is stimulated by SOS3 and SCaBP8, but inhibited by ABI2 and GI. The activated SOS2 protein reduced the inhibition of the K^+^ channel AKT1 by SCaBP8. SOS2 and MAPK6 interact with and phosphorylate PIFs, then promote their rapid degradation to improve salt tolerance. ELF3 enhances salt tolerance by inhibiting *PIF4* transcription and inducing GI degradation, which in turn releases SOS2. Salt stress promotes the transfer of COP1 from the nucleus to the cytoplasm, thereby reducing the degradation of HY5. Under light, HY5 accumulates and interacts with salt-induced HDA9, which enhances the binding ability of HDA9 with the downstream *HsfA2* gene, thereby inhibiting its transcription and increasing salt stress. Meanwhile, the accumulation of HY5 protein directly binds to promoters of *P5CS1* and *ABI5* genes to activate their transcription, which induces proline and ABA accumulation, thereby enhancing plant salt tolerance. In addition, salt stress inhibits *OsBBX17* expression, but activates *MAPK1*. The phosphorylation of OsBBX17 by OsMAPK1 further inhibits its repression of *OsHAK2/7*, promoting ion homeostasis and salt tolerance. Meanwhile, blue light activates NF-Y heterotrimer through CRY. GmNF-Y heterotrimer activates the PYR1-mediated ABA signaling pathway and JA signaling pathway. The salt stress-induced JA synthesis inhibits JAZ8 to prevent the development of NF-Ys trimers. The PRR5, PRR7, and PRR9 are negative regulators in ABA biosynthesis. OsPRR73 binds the promoter of *OsHKT2;1* to enhance salinity stress by recruiting HDAC10. In addition, TaGBF1 is induced by blue light, which confers hypersensitivity to ABA and salt.

Phytohormones are key endogenous regulating molecules in plants that respond to salt stress [119, 120]. They primarily enhance plant tolerance to salt by regulating gene expression to encourage the build-up of substances that maintain osmotic balance, scavenging ROS to maintain oxidative balance, controlling ion channel opening and closing to maintain ionic balance, and modifying plant growth and development [121, 122] (Fig. 3). Salt stress induces a rapid increase in ABA levels, which regulate plant osmosis, ion homeostasis, and ROS [120]. By phosphorylating downstream AREB-ABF transcription factors, the accumulation of endogenous ABA increases the activity of SnRK2s and encourages stomatal closure [123]. Regulation of β-AMYLASE1 (BAM1) and α-AMYLASE3 (AMY3) catabolizes starch as a regulatory substance to regulate osmotic homeostasis [124]. Some studies have shown that salt stress is negatively regulated by phytochrome A in concert with the ABA and JA pathways [116]. After suppressing the *CaPIF8* gene, salt tolerance decreased, while ABA-related gene expression rose dramatically [90]. The ABA signaling pathway also interacts with the SOS system, e.g. the ABA-negative regulator ABI2 deactivated SOS2 in order to adversely affect salt tolerance in *Arabidopsis* [125] (Fig. 4). Under salt stress, JA and ABA behave antagonistically [126]. ZmEREB57 promoted JA biosynthesis and the process is dependent on COI1, thereby improving salt tolerance in maize [127] (Fig. 3). To increase salt tolerance, GmNF-YC14 of the NUCLEAR FACTOR-Y family in soybeans, which is a light signaling factor, combines with GmNF-YA16 and GmNF-YB2 to generate a heterotrimer that activates the PYRABACTIN RESISTANCE 1 (PYR1)-mediated ABA signaling pathway [128] (Fig. 4). On the other hand, the salt stress-induced JA synthesis inhibits the jasmonate negatively regulated factor JASMONATE ZIM-DOMAIN 8 (JAZ8), which prevents the formation of NF-Ys trimers [129]. ABI5 is physically interacting with the key circadian clock proteins PSEUDO-RESPONSE REGULATOR 5 (PRR5) and PRR7, which in turn stimulates *ABI5*'s transcriptional activity to improve ABA signaling and sustain healthy seed germination and postgermination growth [130]. Furthermore, PRR5, PRR7, and PRR9 have a detrimental role in the biosynthesis of ABA [130] (Fig. 4). By enlisting HDAC10, OsPRR73 binds the *OsHKT2;1* promoter and suppresses *OsHKT2;1* expression. It improves salt tolerance by lowering the import of cellular sodium ions and ROS accumulation [131] (Fig. 4).

Under salt stress, ET is also generated in significant numbers, and SOS2 increases CONSTITUTIVE TRIPLE RESPONSE1 (CTR1) phosphorylation inactivation, which promotes ETHYLENE INSENSITIVE 3 (EIN3) accumulation, then improves plants’ salt tolerance [132] (Fig. 3). Salicylic acid (SA) modulates *GLUTATHIONE TRANSFERASES* (*GST*) transcription, producing reactive oxygen scavengers, such as POD, SOD, and CAT, which increase plant tolerance to salt and enhance ion balance by stimulating the activation of ion channels like SODIUM-HYDROGEN EXCHANGER (NHX1)/SOS1 [133] (Fig. 3). Low R/FR-induced BR and PIF-BES1 signaling pathway to promote hypocotyl elongation under salt stress [91] (Fig. 3). DELLA accumulation, brought about by gibberellin (GA) reduction, offsets salt tolerance and improves salt tolerance and growth retardation [134]. When exposed to light, the degradation of PIF-LIKE14 (OsPILl4) raises GA contents, thereby promoting plant growth [134] (Fig. 3). These results highlight that light temperature-regulated factors mediate plant hormone effects on salt stress response.

## The molecular link between light signaling and salt response

Salt stress-induced SOS2 kinase activity is significantly boosted by light-activated photoreceptor phyA and phyB [8] (Fig. 4). In light, photoactivated phytochromes (Pfr) translocate into the nucleus, interact with SOS2, and promote SOS2 kinase activity in response to salt stress [8] (Fig. 4). PIF1 and PIF3, which negatively regulate salt tolerance, are immediately phosphorylated by SOS2 [8]. SOS2 promotes the degradation of PIFs via the 26S proteasome pathway and relieves their suppression on plant salt tolerance [8] (Fig. 4). In addition, PIF4 inhibits plant salt tolerance by regulating the transcription of many salt-responsive genes [8]. ELF3 enhances salt tolerance by inhibiting PIF4 transcription and inducing GI degradation [8], which in turn releases SOS2 [108]. SOS2 interacts with and phosphorylates SOS1 to activate it extruding Na^+^ from the cytosol to the apoplast [108] (Fig. 4).

The expression of *FAR-RED ELONGATED HYPOCOTYL 3/FAR-RED IMPAIRED RESPONSE 1* (*CsFHY3/FAR1*) increased dramatically in the tea plant (*Camellia sinensis*) after exposure to salt stress [135]. Salt stress significantly induces the activity of HISTONE DEACETYLASE 9 (HDA9) [136]. The large accumulation of HY5 in the light enhances its interaction with HDA9, which promotes the binding ability of HY5 to the *HEAT-SHOCK FACTOR A2* (*HsfA2*) promoter [136]. HY5 represses *HsfA2* gene expression with the cooperation of HDA9, thereby increasing salt tolerance [136] (Fig. 4). Additionally, HY5 directly associates with *P5CS1* and engenders a supercomplex to enhance the *P5CS1* activity, leading to a large amount of proline accumulation, thereby promoting the osmotic balance to enhance salt tolerance after exposure to light from dark [137].

Interestingly, the salt tolerance of the *cop1* mutant is restored to the WT after the application of exogenous sucrose, indicating that COP1 positively regulates salt tolerance in *Arabidopsis* and is related to its sucrose content [138]. In addition, COP1 is largely retained in the cytoplasm and promotes the stability of HY5 in the nucleus under salt stress, which enhances the transcriptional activation of HY5 on *ABI5* [74]. However, ethylene facilitates the nuclear import of COP1, counteracting its nucleocytoplasmic shuttling induced by salt stress and establishing a complex regulatory network [65]. Furthermore, PIL14 and SLENDER RICE1 (SLR1) form a transcriptional module that integrates light and GA signals to fine-tune plant growth under salt stress in rice [134]. These results indicate that photoactivated phytochromes positively regulate salt response by directly promoting the SOS2-PIFs module or indirectly modulating the hormones (such as ABA, ethylene, GA, and JA) signaling, proline, and sucrose signaling through COP1-HY5 module or other pathways, thus maximizing the ratio of stress tolerance to plant development.

## Conclusions and future perspectives

In protected vegetable production, due to the blind pursuit of high-yield unscientific fertilization and the long-term lack of rainwater, the degree of soil salinization in the protected vegetable production system is becoming more and more serious, which seriously restricts its yield and quality. Soil undergoes salinization along with changes in physicochemical properties, which results in sticky soil structure, poor permeability, nutrient release, and other issues that create a host of ecological and environmental challenges that worsen surface soil salinization, consolidate the soil, and drastically lower crop yields [139]. Salinity stress significantly reduces the metabolic activity and functional diversity of soil microorganisms, and the decline in soil bacterial community diversity and abundance significantly reduces the utilization of soil carbon sources, further exacerbating soil deterioration [140]. Climate change, which accelerates land degradation and desertification, is another reported major reason for salinization [141]. Given the global imperative to sustainably increase food production against the backdrop of accelerating climate change, we need to accelerate the delivery of outputs from plant salinity tolerance research. For example, applying functional carbon nanodots (FCNs) exogenously can enhance soil fertility, create a beneficial cycle between soil–microbes–plants, boost soil fertility to fix nitrogen, and encourage tomato yield by enhancing nutrient uptake [142].

In addition, recent studies have indicated that light signals are involved in improving salt resistance [1, 8, 131, 134]. Although great leaps and bounds have been achieved in the regulatory networks underlying light-to-salt signaling, we are still in the infancy phase of understanding the molecular mechanism of plant responses to numerous stressors. The better way to improve the resistance of vegetables is to breed new varieties with a stronger tolerance to diverse and increasingly severe environmental stresses. It needs us to make efforts to identify the hypothetical elements of plant reactions toward numerous environmental stresses and elucidate their molecular mechanisms. For example, the positive regulatory factor of salt stress, SOS2, was demonstrated to contribute to plant responses to shade [8]. There are still many significant unanswered questions regarding the functionality of SOS in the circadian clock, plant growth and biomass, hormone signaling and metabolism, and cross-reactivity with other cell-mediated signaling pathways. These questions must be answered to fully characterize the diversity of SOS elements in plants [8]. Regulating the expression timing and cell type specificity of *SOS* genes, as well as gene expression and protein stability, could be an effective approach to engineering salt resistance in crops. Future research should primarily focus on identifying the light-signaling-regulated E3 ligases that facilitate the ubiquitination of SOS core proteins. Understanding mechanisms of light signaling that regulate histone marks on chromatin regions of the main genes of the SOS pathway will help substantially increase salinity tolerance in existing crops using genetic modification or genome editing.

Furthermore, exploration of Na^+^-sensing upstream pathways, which are regulated by light signaling, might help us to comprehend the unique salinity response systems in plants. These potentially enable plant scientists to establish a salt stress regulation network in plants exclusion of the classic SOS system, as well as providing theoretical and technical support for the control light environment to regulate the salt tolerance of vegetables in a protected cultivation system. Nonetheless, the balance between plant growth and resistance is an eternal topic. How to balance the relationship between plant growth and salt resistance using LED technology remains to be further explored. In addition, how to precisely control the light spectrum to increase the efficiency of light energy usage for crop quality and production under saline–alkali environments still needs to be clarified in the future.

## Data Availability

No data was used for the research described in the article.
